# Construction and evaluation of a nomogram prediction model for aspiration pneumonia in patients with acute ischemic stroke

**DOI:** 10.1016/j.heliyon.2023.e22048

**Published:** 2023-11-08

**Authors:** Junming Wang, Yuntao Wang, Pengfei Wang, Xueting Shen, Lina Wang, Daikun He

**Affiliations:** a Department of General Practice, Jinshan Hospital, Fudan University, Shanghai, 201508, China; b Department of General Practice, Zhongshan Hospital, Fudan University, Shanghai, 200032, China; c Center of Emergency and Critical Care Medicine, Jinshan Hospital, Fudan University, Shanghai, 201508, China; d Research Center for Chemical Injury, Emergency and Critical Medicine of Fudan University, Shanghai, 201508, China; e Key Laboratory of Chemical Injury, Emergency and Critical Medicine of Shanghai Municipal Health Commission, Shanghai, 201508, China

**Keywords:** Nomogram prediction model, Aspiration pneumonia, Acute ischemic stroke, Risk factors

## Abstract

**Background:**

Aspiration Pneumonia (AP) is a leading cause of death in patients with Acute Ischemic Stroke (AIS). Early detection, diagnosis and effective prevention measures are crucial for improving patient prognosis. However, there is a lack of research predicting AP occurrence after AIS. This study aimed to identify risk factors and develop a nomogram model to determine the probability of developing AP after AIS.

**Method:**

A total of 3258 AIS patients admitted to Jinshan Hospital of Fudan University between January 1, 2016, and August 20, 2022, were included. Among them, 307 patients were diagnosed with AP (AP group), while 2951 patients formed the control group (NAP group). Univariate and multivariate logistic regression analyses were conducted to identify relevant risk factors for AP after AIS. These factors were used to establish a scoring system and develop a nomogram model using R software.

**Results:**

Univariate analysis revealed 20 factors significantly associated (P < 0.05) with the development of AP after AIS. These factors underwent multivariate logistic regression analysis, which identified age (elderly), National Institute of Health Stroke Scale (NIHSS) score, dysphagia, atrial fibrillation, cardiac insufficiency, renal insufficiency, hepatic insufficiency, elevated Fasting Blood Glucose (FBG), elevated C-Reactive Protein (CRP), elevated Neutrophil percentage (NEUT%), and decreased prealbumin as independent risk factors. A nomogram model incorporating these 11 risk factors was constructed, with a C-index of 0.872 (95 % CI: 0.845–0.899), indicating high accuracy. Calibration and clinical decision analyses demonstrated the model's reliability and clinical value.

**Conclusion:**

A nomogram model incorporating age, NIHSS score, dysphagia, atrial fibrillation, cardiac insufficiency, renal insufficiency, hepatic insufficiency, FBG, CRP, NEUT%, and prealbumin effectively predicts AP risk in AIS patients. This model provides guidance for early intervention strategies, enabling the identification of high-risk individuals for timely preventive measures.

## 1 Introduction

Acute Ischemic Stroke (AIS) is the most common type of stroke, accounting for approximately 70 % of all stroke cases in China [1]. As living standards improve and lifestyles change, China has witnessed a significant increase in the number of stroke cases, making it the country with the highest stroke burden globally [2]. Stroke remains a leading cause of death and disability, ranking second and third, respectively. Alarmingly, there is a growing trend of stroke occurring in younger individuals, posing a significant threat to public health [3]. Aspiration Pneumonia (AP), a common complication following a stroke, is strongly associated with poor patient outcomes [4,5]. Accumulating evidence indicates that risk factors such as dysphagia and the immunosuppressive status resulting from stroke can increase susceptibility to AP and mortality rates [6]. Consequently, early prevention and treatment of AP following a stroke have become crucial areas of clinical focus. The ability to identify independent risk factors and accurately predict individuals at a high risk of developing AP after a stroke would allow for effective interventions at an early stage, ultimately improving patient prognosis and quality of life.

In recent years, numerous clinical studies addressing stroke-associated pneumonia have been conducted worldwide. Building upon these studies, the Pneumonia in Stroke Consensus Group (PISCES) released the "Recommendations on Diagnosis of Stroke-Associated Pneumonia" in 2015 and developed a predictive model for SAP [7]. Impaired consciousness, dysphagia, and immune dysfunction resulting from stroke are considered pivotal pathogenic factors for SAP [8,9], similarly contributing mechanisms to the development of AP after AIS [10,11]. In addition to cerebral infarction, other conditions like Chronic Obstructive Pulmonary Disease (COPD), Parkinson's Disease (PD) and epilepsy have also been reported as important underlying causes of AP [[12], [13], [14]], but the correlation between clinical features in patients with specific diseases and the risk of AP has rarely been studied.

Previous clinical studies have suggested that advanced age, impaired consciousness, dysphagia, and comorbid conditions (e.g. COPD and atrial fibrillation) are independent risk factors for AP in stroke patients [[15], [16], [17], [18]]. Nevertheless, few studies have comprehensively assessed the synergistic effect of multiple risk factors on increasing the risk of AP after a stroke using models like nomograms, which currently fall short of meeting clinical demands. Nomograms, constructed using logistic regression models, are graphical tools comprising line segments representing various predictors. They possess several advantages, such as user-friendliness, easy interpretability, and effective predictive capabilities, contributing to their growing adoption in clinical practice [19].

Therefore, our subject was to identify factors independently influencing the development of AP in post-AIS patients. Subsequently, we established a predictive nomogram model based on comprehensive clinical data from AIS-diagnosed patients in a tertiary hospital. We aimed to provide a reference for reducing the incidence of AP and improving patient outcomes.

## 2 Material and methods

### 2.1 Patients

Our study included 3258 AIS patients (1953 males, 1305 females) admitted to Jinshan Hospital of Fudan University between January 1, 2016, and August 20, 2022, who met the inclusion and exclusion criteria. All patients were treated according to the "Chinese Guidelines for the Diagnosis and Treatment of Acute Ischemic Stroke" (version 2014 was applied before 2018 and version 2018 was applied after that) after admission [20]. They were divided into AP and NAP groups according to whether AP occurred during hospitalization. There were 307 patients in the AP group, aged 79 (72, 85) years, including 156 males. Other 2951 patients were classified as the NAP group, aged 69 (61, 78) years, of whom 1797 were male. The entire cohort (n = 3258) was randomly split into a training cohort (n = 2281) and a validation cohort (n = 977) at a 7:3 ratio.

The criteria for diagnosing Acute Ischemic Stroke (AIS) are outlined in the "Chinese Guidelines for the Diagnosis and Treatment of Acute Ischemic Stroke" (version 2014 was applied before 2018 and version 2018 was applied after that). The criteria include sudden onset of symptoms such as weakness or numbness on one side of the body, language difficulties, and imaging evidence of brain lesions that persist for more than 24 h. Other potential causes must be excluded, and imaging must show no signs of cerebral hemorrhage. Diagnosis must be made by two experienced neurology professionals with at least five years of clinical and scientific research experience.

The diagnostic criteria for aspiration pneumonia (AP) are outlined in the "Guideline of Diagnosis and Treatment of Adults with Community-acquired Pneumonia" [21] released by the American Thoracic Society and Infectious Diseases Society of America. To diagnose AP, there must be evidence of both risk factors and aspiration, as well as a basis for diagnosing pneumonia. It is recommended that clinical diagnosis be performed by two experienced physicians who have been actively involved in researching and treating AP for at least 5 years.

### 2.2 Inclusion and exclusion criteria

Inclusion criteria: ①Patients with AIS confirmed by cranial imaging examination and meeting diagnostic criteria; ②The first cranial CT scan of the patient after admission showed no cerebral hemorrhage; ③First onset; ④Clinical data must be complete.

Exclusion criteria: ①Patients with incomplete clinical information or data; ②More than 7 days from onset to hospitalization; ③Recent history of significant trauma to the head, gastrointestinal bleeding and abdominal surgery; ④History of fever or infection within nearly 2 weeks.

### 2.3 Extraction of baseline data

After receiving ethical approval (Research Ethics Committee of Jinshan Hospital, Fudan University, China, Ref.-No. JIEC 2023-S34), demographic data and disease-related data screened from clinical case files were extracted from the internal database of Jinshan Hospital, including case number, primary diagnosis, AP, age, gender, National Institute of Health Stroke Scale (NIHSS) score, dysphagia, encephalanalosis, hypertension, diabetes, hyperlipidemia, COPD, Coronary Heart Disease (CHD), atrial fibrillation, cardiac insufficiency, renal insufficiency, hepatic insufficiency, Fasting Blood Glucose (FBG), C-Reactive Protein (CRP), White Blood Cell count (WBC), Neutrophil percentage (NEUT%), Hemoglobin (HB), Platelet count (PLT), albumin, prealbumin, triglyceride, Total Cholesterol (TC), Low-Density Lipoprotein (LDL), homocysteine, Potassium (K^+^) and Sodium (Na^+^). Underlying diseases like encephalanalosis, hypertension, diabetes, hyperlipidemia, COPD, CHD, atrial fibrillation, cardiac insufficiency, renal insufficiency and hepatic insufficiency were obtained from the International Classification of Diseases-10 (ICD-10) number on the medical record Homepage. All the results were the first examination results after admission. After data washing, they were deposited into an Excel database.

### 2.4 Determination of data

AIS: In accordance with the diagnostic criteria outlined in the "Chinese Guidelines for the Diagnosis and Treatment of Acute Ischemic Stroke" (version 2014 was applied before 2018 and version 2018 was applied after that), acute ischemic stroke is defined as the presence of clinical symptoms and signs, accompanied by a new lesion confirmed by skull CT or MRI, with a duration of ≤7 days. NIHSS score: The NIHSS score was employed to gauge the degree of neurological impairment in patients, with ≤5 as mild, 6 to 20 as moderate, and >20 as severe [22]. Evaluation of swallowing disorders was performed by the bedside water swallow test [23]: the patient took an end sitting position and drank 30 ml of warm and boiled water. Observe the time needed for drinking and choking situations. The grading standards are as follows: grade 1: Patients didn't choke in 5s and can swallow in once; Grade 2: Patients didn't choke in 5–10s but need to swallow for 2 times; Grade 3: Patients can swallow in 5–10s but there was choking; Grade 4: Patients need to swallow for more than 2 times in 5–10s and there was choking; Grade 5; Patients can't swallow in 10s and multiple choking episodes [24]. The results were initially assessed by nurses and subsequently reviewed by specialized doctors in the neurology department of Jinshan Hospital. The grade ≥3 on the drinking test was judged as dysphagia. For those with grade = 2, an additional Videofluoroscopic Swallowing Study (VFSS) was applied to clarify swallowing disorders. Encephalanalosis: The diagnoses are jointly discussed and made by two imaging physicians based on brain CT images during the examination in Jinshan Hospital. In cases of disagreement, they were ultimately determined by the higher-level physician.

### 2.5 Statistical analysis

Statistical analysis was conducted using SPSS software (version 26.0) and R software (version 4.2.3). We used the number of cases or percentages to represent categorical data and applied the chi-square test for analysis. Continuous variables were represented by M (P_25_, P_75_) and a nonparametric test for two independent samples was used. We used logistic stepwise regression to identify the factors that influenced AP in patients with AIS. A statistically significant difference was determined using a significance level (α) of 0.05. The multiple regression model was constructed using the R software package "MASS", employing stepwise regression. The Akaike Information Criterion (AIC) value was utilized to evaluate the model. Based on the results of multiple factor regression, a nomogram prediction model for AP after AIS was established using the R software package "rms". The stacked histogram, box plot and PCA plot were established using the R software package "ggplot 2". The heatmap was established using the R software package "pheatmap" and Canberra distance was used during clustering. To evaluate the accuracy of the nomogram model, we used several methods such as calibration charts, Decision Curve Analysis (DCA), and C-index. We also validated the model using the bootstrap method with 1000 repetitions. Finally, we assessed the clinical effectiveness of the model by analyzing the clinical impact curve.

## 3 Results

### 3.1 Descriptive and other characteristics of participants in training and validation group

The information pertaining to the baseline clinical characteristics of both the training and validation groups is presented in Table 1. Our study included 3258 patients with a history of AIS, randomly divided into a 7:3 ratio training group (2281 patients) and a validation group (977 patients). No significant differences were found (P > 0.05) in terms of age, gender, NIHSS score, dysphagia, encephalanalosis, hypertension, diabetes, hyperlipidemia, COPD, CHD, atrial fibrillation, cardiac insufficiency, renal insufficiency, hepatic insufficiency, FBG, CRP, WBC, NEUT%, HB, PLT, albumin, prealbumin, triglyceride, TC, LDL, homocysteine, K^+^ and Na^+^ between two groups. The incidence rates of AP were 9.6 % in the training group and 8.9 % in the validation group.

**Table 1:** Descriptive and other characteristics of participants in training and validation group.

Variables	All	Training group				Validation group				P^#^
		Total (n = 2281)	NAP (n = 2061)	AP (n = 220)	P^$^	Total (n = 977)	NAP (n = 890)	AP (n = 87)	P^$^	
**Age [Years, M(P25,P75)]**	71 (62.79)	71 (62, 79)	70 (62, 78)	79 (73, 85)	＜0.001***	71 (61, 79)	69 (61, 79)	79 (71, 85)	＜0.001***	0.632
**Age (Years, %)**										
≤44	88 (2.7)	60 (2.6)	59 (2.9)	1 (0.5)	＜0.001***	28 (2.9)	26 (2.9)	2 (2.3)	＜0.001***	0.681
45-59	588 (18.0)	401 (17.6)	390 (18.9)	11 (5.0)		187 (19.1)	183 (20.6)	4 (4.6)		
60-74	1368 (42.0)	969 (42.5)	911 (44.2)	58 (26.4)		399 (40.8)	374 (42.0)	25 (28.7)		
75-89	1126 (34.6)	793 (34.8)	658 (31.9)	135 (61.4)		333 (34.1)	287 (32.2)	46 (52.9)		
≥90	88 (2.7)	58 (2.5)	43 (2.1)	15 (6.8)		30 (3.1)	20 (2.2)	10 (11.5)		
**Gender (%)**										
Male	1953 (59.9)	1359 (59.6)	1248 (60.6)	111 (50.5)	0.004**	594 (60.8)	549 (61.7)	45 (51.7)	0.069	0.515
Female	1305 (40.1)	922 (40.4)	813 (39.4)	109 (49.5)		383 (39.2)	341 (38.3)	42 (48.3)		
**NIHSS score [n, M(P25,P75)]**	1 (0, 3)	1 (0, 3)	1 (0, 3)	3 (1, 12)	＜0.001***	1 (0, 3)	1 (0, 3)	6 (2, 15)	＜0.001***	0.667
**NIHSS score (n, %)**										
≤5	2755 (84.6)	1936 (84.9)	1808 (87.7)	128 (58.2)	＜0.001***	819 (83.8)	778 (87.4)	41 (47.1)	＜0.001***	0.736
6-20	432 (13.3)	297 (13.0)	231 (11.2)	66 (30.0)		135 (13.8)	102 (11.5)	33 (37.9)		
＞20	71 (2.2)	48 (2.1)	22 (1.1)	26 (11.8)		23 (2.4)	10 (1.1)	13 (14.9)		
**Dysphagia (Yes, %)**	161 (4.9)	111 (4.8)	71 (3.4)	40 (18.2)	＜0.001***	50 (5.1)	33 (3.7)	17 (19.5)	＜0.001***	0.762
**Encephalanalosis (Yes, %)**	773 (23.7)	529 (23.1)	467 (22.7)	62 (28.2)	0.065	244 (24.9)	225 (25.3)	19 (21.8)	0.479	0.273
**Hypertension (Yes, %)**	2526 (77.5)	1787 (78.3)	1618 (78.5)	169 (76.8)	0.564	739 (75.6)	675 (75.8)	64 (73.6)	0.636	0.090
**Diabetes (Yes, %)**	1084 (33.3)	753 (33.0)	676 (32.8)	77 (35.0)	0.509	331 (33.8)	303 (34.0)	28 (32.2)	0.726	0.630
**Hyperlipidemia (Yes, %)**	753 (23.1)	539 (23.6)	505 (24.5)	34 (15.5)	0.003**	214 (21.9)	206 (23.1)	8 (9.2)	0.003**	0.284
**COPD (Yes, %)**	91 (2.8)	65 (2.8)	52 (2.5)	13 (5.9)	0.004**	26 (2.6)	18 (2.0)	8 (9.2)	＜0.001***	0.765
**CHD (Yes, %)**	347 (10.7)	252 (11.0)	212 (10.3)	40 (18.2)	＜0.001***	95 (9.7)	87 (9.8)	8 (9.2)	0.862	0.262
**Atrial fibrillation (Yes, %)**	390 (12.0)	276 (12.0)	201 (9.8)	75 (34.1)	＜0.001***	114 (11.6)	86 (9.7)	28 (32.2)	＜0.001***	0.728
**Cardiac insufficiency (Yes, %)**	201 (6.2)	143 (6.2)	97 (4.7)	46 (20.9)	＜0.001***	58 (5.9)	46 (5.2)	12 (13.8)	0.001**	0.718
**Renal insufficiency (Yes, %)**	177 (5.4)	126 (5.5)	96 (4.7)	30 (13.6)	＜0.001***	51 (5.2)	37 (4.2)	14 (16.1)	＜0.001***	0.726
**Hepatic insufficiency (Yes, %)**	242 (7.4)	170 (7.4)	136 (6.6)	34 (15.5)	＜0.001***	72 (7.3)	60 (6.7)	12 (13.8)	0.016*	0.934
**FGB [mmol/L, M(P25,P75)]**	5.59 (4.98, 6.93)	5.57 (4.96, 6.92)	5.53 (4.94, 6.75)	6.48 (5.23, 8.22)	＜0.001***	5.64 (5.03, 6.96)	5.61 (5.02, 6.83)	6.24 (5.04, 8.15)	＜0.001***	0.231
**FGB (mmol/L, %)**										
4-7	2419 (74.2)	1693 (70.0)	1561 (75.7)	132 (60.0)	＜0.001***	726 (74.3)	672 (75.5)	54 (62.1)	0.006**	0.958
＜4 or ＞7	839 (25.8)	588 (30)	500 (24.3)	88 (40.0)		251 (25.7)	218 (24.5)	33 (37.9)		
**CRP [mg/L, M(P25,P75)]**	1.44 (0.50, 5.20)	1.47 (0.50, 5.19)	1.26 (0.50, 3.91)	13.53 (3.72, 45.60)	＜0.001***	1.29 (0.50, 5.21)	1.16 (0.50, 3.58)	13.8 (2.73, 36.62)	＜0.001***	0.310
**CRP (mg/L, %)**										
＜10	2733 (83.9)	1913 (83.9)	1818 (88.2)	95 (43.2)	＜0.001***	820 (83.9)	779 (87.5)	41 (47.1)	＜0.001***	0.307
10-19	220 (6.8)	143 (6.3)	110 (5.3)	33 (15.0)		77 (7.9)	63 (7.1)	14 (16.1)		
20-29	92 (2.8)	65 (2.8)	48 (2.3)	17 (7.7)		27 (2.8)	21 (2.4)	6 (6.9)		
30-39	55 (1.7)	41 (1.8)	29 (1.4)	12 (5.5)		14 (1.4)	8 (0.9)	6 (6.9)		
40-49	44 (1.4)	31 (1.4)	17 (0.8)	14 (6.4)		13 (1.3)	6 (0.7)	7 (8.0)		
≥50	114 (3.5)	88 (3.9)	39 (1.9)	49 (22.3)		26 (2.7)	13 (1.5)	13 (14.9)		
**WBC [×10**^**9**^**/L, M(P25,P75)]**	6.1 (5.0, 7.5)	6.1 (5.0, 7.5)	6.0 (4.9, 7.3)	8.0 (5.7, 10.4)	＜0.001***	6.2 (5.1, 7.6)	6.1 (5.0, 7.4)	7.1 (5.5, 9.7)	＜0.001***	0.318
**WBC (×10**^**9**^**/L, %)**										
4-10	2791 (85.7)	1944 (85.2)	1795 (87.1)	149 (67.7)	＜0.001***	847 (86.7)	782 (87.9)	65 (74.7)	＜0.001***	0.273
＜4 or ＞10	467 (14.3)	337 (14.8)	266 (12.9)	71 (32.3)		130 (13.3)	108 (12.1)	22 (25.3)		
**NEUT% [%, M(P25,P75)]**	62.8 (56.2, 70.3)	62.6 (56.1, 70.3)	61.8 (55.6, 68.4)	77.7 (70.6, 83.3)	＜0.001***	63.2 (56.6, 70.2)	62.3 (55.8, 68.9)	74.7 (67.7, 81.3)	＜0.001***	0.744
**NEUT% (%, %)**										
＜80	3016 (92.6)	2107 (92.4)	1968 (95.5)	139 (63.2)	＜0.001***	909 (93.0)	847 (95.2)	62 (71.3)	＜0.001***	0.505
≥80	242 (7.4)	174 (7.6)	93 (4.5)	81 (36.8)		68 (7.0)	43 (4.8)	25 (28.7)		
**HB [g/L, M(P25,P75)]**	132 (121, 143)	132 (121, 143)	132 (121, 143)	127 (114, 138)	＜0.001***	133 (121, 143)	133 (122, 144)	127 (111, 142)	＜0.001***	0.367
**HB (g/L, %)**										
≥120	2529 (77.6)	1767 (77.5)	1623 (78.7)	144 (65.5)	＜0.001***	762 (78.0)	705 (79.2)	57 (65.5)	0.009**	0.893
119-90	667 (20.5)	469 (20.6)	411 (19.9)	58 (26.4)		198 (20.3)	172 (19.3)	26 (29.9)		
89-60	56 (1.7)	40 (1.8)	24 (1.2)	16 (7.3)		16 (1.6)	12 (1.3)	4 (4.6)		
＜60	6 (0.2)	5 (0.2)	3 (0.1)	2 (0.9)		1 (0.1)	1 (0.1)	0 (0.0)		
**PLT [×10**^**9**^**/L, M(P25,P75)]**	195 (157, 236)	195 (158, 236)	196 (159, 237)	187 (148, 230)	0.053	193 (156, 236)	195 (159, 237)	170 (137, 223)	0.016*	0.847
**Albumin [g/L, M(P25,P75)]**	37 (35, 39)	37 (35, 39)	37 (35, 39)	35 (32, 37)	＜0.001***	37 (35, 39)	37 (35, 40)	35 (32, 38)	＜0.001***	0.055
**Albumin (g/L, %)**										
≥40	693 (21.3)	460 (20.2)	431 (20.9)	29 (13.2)	＜0.001***	233 (23.8)	223 (25.1)	10 (11.5)	＜0.001***	0.061
39-30	2452 (75.3)	1742 (76.4)	1580 (76.7)	162 (73.6)		710 (72.7)	644 (72.4)	66 (75.9)		
＜30	113 (3.5)	79 (3.5)	50 (2.4)	29 (13.2)		34 (3.5)	23 (2.6)	11 (12.6)		
**Prealbumin [mg/L, M(P25,P75)]**	228 (185, 270)	227 (185, 269)	233 (192, 272)	164 (122, 205)	＜0.001***	228 (184, 271)	233 (193, 275)	165 (127, 200)	＜0.001***	0.439
**Prealbumin (mg/L, %)**										
≥180	2524 (77.5)	1770 (77.6)	1769 (85.8)	91 (41.4)	＜0.001***	754 (77.2)	720 (80.9)	34 (39.1)	＜0.001***	0.661
179-150	337 (10.3)	227 (10.0)	184 (8.9)	43 (19.5)		110 (11.3)	89 (10.0)	21 (24.1)		
149-100	315 (9.7)	225 (9.9)	163 (7.9)	62 (28.2)		90 (9.2)	71 (8.0)	19 (21.8)		
＜100	82 (2.5)	59 (2.6)	35 (1.7)	24 (10.9)		23 (2.4)	10 (1.1)	13 (14.9)		
**Triglyceride [mmol/L, M(P25,P75)]**	1.27 (0.96, 1.77)	1.27 (0.95, 1.78)	1.29 (0.97, 1.83)	1.11 (0.82, 1.42)	0.001**	1.27 (0.96, 1.77)	1.30 (0.97, 1.80)	1.05 (0.82, 1.30)	0.004**	0.820
**Triglyceride (mmol/L, %)**										
0.56–1.7	2299 (70.6)	1614 (70.8)	1439 (69.8)	175 (79.5)	0.003**	685 (70.1)	614 (69.0)	71 (81.6)	0.014*	0.711
＜0.56 or ＞1.7	959 (29.4)	667 (29.2)	622 (30.2)	45 (20.5)		292 (29.9)	276 (31.0)	16 (18.4)		
**TC [mmol, M(P25,P75)]**	4.35 (3.62, 5.14)	4.34 (3.60, 5.14)	4.34 (3.61, 5.14)	4.45 (3.5, 5.24)	0.545	4.36 (3.67, 5.11)	4.37 (3.68, 5.16)	4.26 (3.59, 4.66)	0.062	0.616
**LDL [mmol/L, M(P25,P75)]**	2.78 (2.22, 3.37)	2.78 (2.20, 3.38)	2.78 (2.21, 3.38)	2.72 (2.11, 3.40)	0.361	2.77 (2.25, 3.35)	2.78 (2.26, 3.40)	2.70 (2.15, 3.07)	0.069	0.820
**Homocysteine [μmol/L, M(P25,P75)]**	13.4 (10.8, 17.2)	13.3 (10.8, 17.1)	13.2 (10.8, 17.1)	13.9 (10.9, 17.6)	0.299	13.5 (10.8, 17.5)	13.4 (10.8 17.3)	14.8 (11.7, 19.6)	0.169	0.616
**K** + **[mmol/L,M(P25,P75)]**	3.73 (3.49, 3.97)	3.73 (3.50, 3.97)	3.74 (3.51, 3.97)	3.67 (3.39, 3.90)	＜0.001***	3.74 (3.48, 3.98)	3.75 (3.50, 3.98)	3.64 (3.45, 3.90)	0.241	0.769
**K**^**+**^**(mmol/L, %)**										
3.5–5.0	2438 (74.8)	1713 (75.1)	1573 (76.3)	140 (63.6)	＜0.001***	725 (74.2)	668 (75.1)	57 (65.5)	0.052	0.591
＜3.5 or ＞5.0	820 (25.2)	568 (24.9)	488 (23.7)	80 (36.4)		252 (25.8)	222 (24.9)	30 (34.5)		
**Na** + **[mmol/L,M(P25,P75)]**	142 (140, 143)	142 (140, 143)	142 (140, 143)	142 (139, 144)	0.771	142 (140, 143)	142 (140, 143)	142 (139, 143)	0.208	0.977

^#^Represents p-value for group comparison of training group and validation group.

^$^Represents p-value for group comparison of participants with and without AP in training group or validation group.

*Represents P < 0.05; **Represents P < 0.01; ***Represents P < 0.001.

Univariate analysis revealed significant differences (P < 0.05) in 20 baseline clinical characteristics, including age, gender, NIHSS score, dysphagia, hyperlipidemia, COPD, CHD, atrial fibrillation, cardiac insufficiency, renal insufficiency, hepatic insufficiency, FBG, CRP, WBC, NEUT%, HB, albumin, prealbumin, triglycerides and K^+^ between two groups, which were suspected influencing factors for AP in patients with AIS history.

### 3.2 Independent risk factors of AP after AIS

To further elucidate the role of relevant factors in the occurrence of AP in patients with AIS, a linear regression model was constructed using 20 baseline clinical characteristics that exhibited statistical differences among the aforementioned factors as independent variables. The assignment of independent variables can be found in Supplementary Table 1. After performing stepwise regression analysis and excluding non-statistically significant independent variables, the results revealed that when the following 13 factors were included in the linear regression model: age, NIHSS score, dysphagia, COPD, atrial fibrillation, cardiac insufficiency, renal insufficiency, hepatic insufficiency, FBG, CRP, WBC, NEUT% and prealbumin, the AIC value reached its lowest point at 201,119. At the current stage, the model's R-squared value is 0.276. By excluding two non-statistically significant variables, the remaining 11 factors (P < 0.05) were associated with the occurrence of AP in patients with AIS, as shown in Table 2.

**Table 2:** Multivariate analysis of the influencing factors of AP in patients with AIS.

Variables	B	SE	Wald	P	OR (95 % CI)
Age	0.551	0.122	20.366	<0.001***	1.735 (1.370–2.212)
NIHSS score	0.581	0.156	13.797	<0.001***	1.787 (1.312–2.423)
Dysphagia	0.642	0.281	5.209	0.022*	1.900 (1.085–3.271)
COPD	0.488	0.390	1.563	0.211	1.628 (0.730–3.392)
Atrial fibrillation	0.417	0.216	3.726	0.007**	1.517 (0.987–2.305)
Cardiac insufficiency	0.488	0.259	3.561	0.001**	1.630 (0.973–2.687)
Renal insufficiency	0.778	0.273	8.100	0.004**	2.177 (1.256–3.677)
Hepatic insufficiency	0.819	0.265	9.558	0.002**	2.269 (1.333–3.775)
FBG	0.468	0.185	6.440	0.011*	1.597 (1.108–2.286)
CRP	0.056	0.058	35.755	<0.001***	1.415 (1.263–1.586)
WBC	0.025	0.215	1.887	0.170	1.343 (0.874–2.031)
NEUT%	0.198	0.232	26.691	<0.001***	3.317 (2.098–5.216)
Prealbumin	0.028	0.103	8.411	0.004**	1.350 (1.100–1.651)

*Represents P < 0.05; **Represents P < 0.01; ***Represents P < 0.001.

### 3.3 Clinical characteristics of people with AP after AIS

**Fig. 1:**
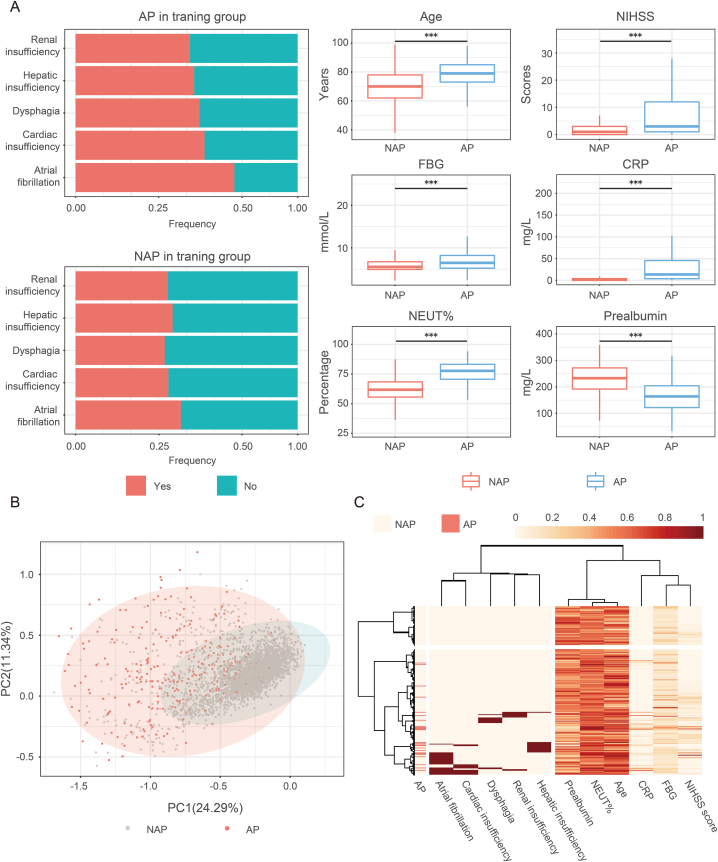
Stack histograms and box plots showed the distribution of independent risk factors selected above in patients from AP and NAP groups. (A) PCA analysis based on the independent risk factors selected above. (B) A heatmap was made of the independent risk factors selected above and clustering of patients and factors was conducted (C). *Represents P < 0.05; **Represents P < 0.01; ***Represents P < 0.001.

There were significant differences in selected independent risk factors between patients from the AP and NAP groups in the training cohort (Fig. 1A). These risk factors were linearly projected to values ranging from 0 to 1. After that PCA analysis was made (Fig. 1B). The result showed that the clinical characteristics of patients from the AP group have significantly changed compared to those from the NAP group in the training cohort. As shown in the heat map (Fig. 1C), patients were classified into 2 clusters based on the selected independent risk factors. Patients with AP after AIS were concentrated in one group.

### 3.4 The construction of the nomogram prediction model

**Fig. 2:**
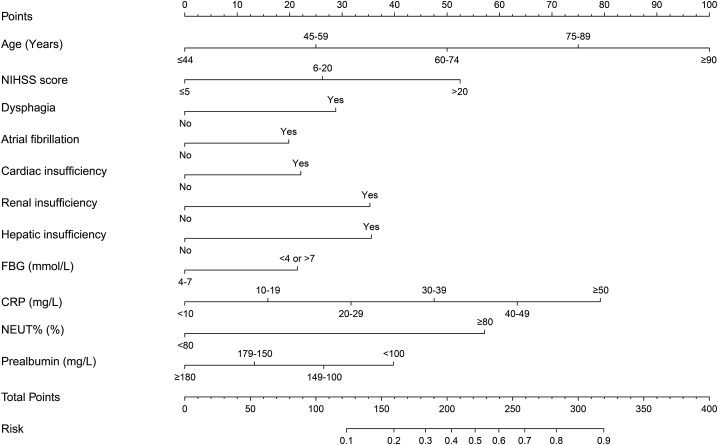
A nomogram prediction model for the risk of AP in patients with AIS history.

A nomogram model was constructed based on 11 risk prediction indicators, including age, NIHSS score, dysphagia, atrial fibrillation, cardiac insufficiency, renal insufficiency, hepatic insufficiency, FBG, CRP, NEUT% and prealbumin. The "Points" above the model represented the corresponding score for each risk factor. This model can personally calculate the corresponding score for each independent risk factor and then ultimately calculate the "Total Points". The "Risk" value corresponding to the "Total Points" downwards was the predicted probability of AP in patients with AIS history, as shown in Fig. 2.

### 3.5 Predictive ability evaluation of the nomogram model

**Fig. 3:**
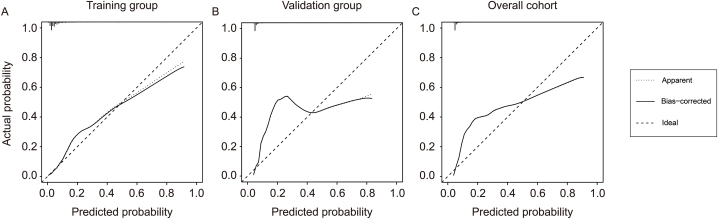
The correction curves between predicted and actual results of the nomogram prediction model for the risk of AP after AIS in the training group (A), validation group (B), and overall cohort (C).

The Nomogram model was evaluated for its predictive ability using 3-fold cross-validation and 1000 iterations. The C-index [equivalent to AUC, the area under the Receiver Operating Characteristic (ROC) curve] of this model was calculated to be 0.872 (95 % CI: 0.845–0.899). The correction curve indicates a good consistency between the predicted risk and actual risk of AP in patients with AIS history (Fig. 3A). The nomogram prediction model had a C-index of 0.847 (95 % CI: 0.800–0.894) in the validation group and 0.865 (95 % CI: 0.841–0.889) in the overall cohort. Fig. 3B and **C** demonstrate good consistency between predicted and actual risk in the validation group and overall cohort, respectively. This suggests that the nomogram prediction model is highly accurate.

### 3.6 Clinical decision evaluation of the nomogram model

**Fig. 4:**
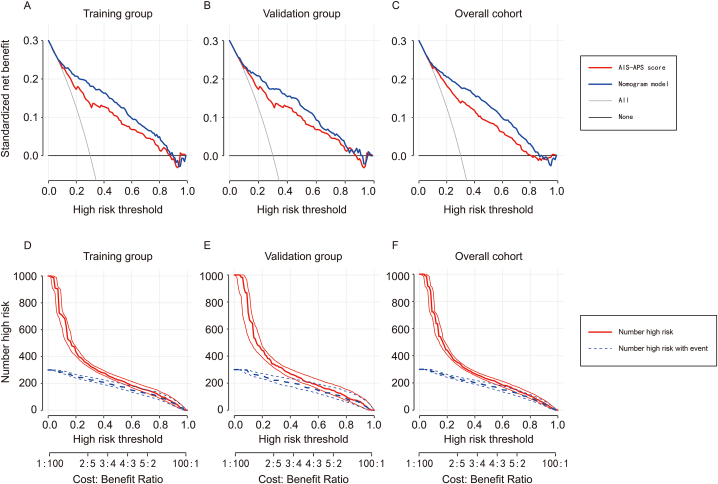
A–C: Clinical decision analysis curve of nomogram model and AIS-APS score in the training group (A), validation group (B) and overall cohort (C); D–F: Clinical impact curves of nomogram model in the training group (D), validation group (E), and overall cohort (F).

A clinical decision analysis (DCA) was made using the high-risk threshold probability as the horizontal axis and the net benefit rate as the vertical axis. The high-risk threshold probability is set from 0 to 1. The black solid line represents the net benefit rate assuming none of the patients develop AP (negative), while the gray solid line represents the rate assuming all patients develop AP (positive). In our study, the blue line represented the decision curve for the nomogram model. The red line was the decision curve of the Acute Ischemic Stroke Associated Pneumonia Score (AIS-APS) recommended by the Chinese Expert Consensus (version 2019) [25] for diagnosing and treating stroke-associated pneumonia. The DCA curve indicated that the nomogram model could provide clinical net benefits to patients in the training group (Fig. 4A), validation group (Fig. 4B), and overall cohort (Fig. 4C) when the threshold probabilities ranged from 0.08 to 0.90, 0.12–0.88, and 0.10–0.81 respectively. Moreover, compared to the AIS-APS score, the column chart showed more net benefits, which can better predict the risk of AP after AIS.

**Fig. 5:**
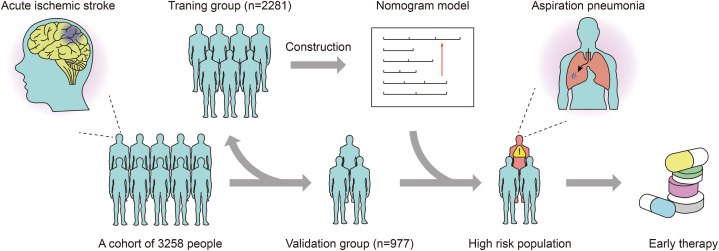
Flowchart displaying the process of building the nomogram.

We created clinical impact curves (Fig. 4D, E, and 4F) based on the DCA curve to assess the effectiveness of the nomogram model. These curves show the number of patients (out of 1000) who may develop AP after AIS in the training group, validation group, and overall cohort. The results showed that when the threshold probability was greater than 20 % of the predicted score probability value, the high-risk population of pneumonia judged by the predictive model was well matched with the actual population of pneumonia, which confirmed that the clinical efficacy of our nomogram predictive model was extremely high. The process for building the nomogram is presented as a flowchart in Fig. 5.

## 4 Discussion

Patients with AIS history have been shown to have multiple risk factors for AP [26]. On the flip side, AIS patients are 4.07 times more likely to die if they have AP [6], making it the main cause of death in those patients [27]. Early identification of AP complicated by AIS and effective anti-infection treatment can significantly improve the prognosis of patients [28]. Therefore, developing a practical and effective risk prediction model for early diagnosis and prognosis evaluation of AP after AIS has become the key to preventing and treating the disease. However, there are few predictive models (such as nomogram models) that can be applied in clinical practice. To investigate the risk factors for AP in AIS patients and aid early targeted treatment, our study retrospectively analyzed a clinical cohort of 3258 and created a nomogram model with significant clinical value.

Our study found that the incidence of AP was 9.42 %, indicating a higher occurrence of AP after AIS, which needed more clinical attention. We used difference testing and multivariate logistic regression analysis to identify independent risk factors for AP in AIS patients. Our results indicated that: age (Elderly), NIHSS score (High), dysphagia (Yes), atrial fibrillation (Yes), cardiac insufficiency (Yes), renal insufficiency (Yes), hepatic insufficiency (Yes), FBG (Abnormal), CRP (Upregulated), NEUT% (Upregulated) and prealbumin (Downregulated) were independent risk factors for the occurrence of AIS associated AP. The possible reason for them might be: (1) **Age**: Based on the findings of a multicenter retrospective survey conducted in northern Japan, it was observed that 79 % of patients diagnosed with AP in emergency care hospitals were aged over 70 years old [29]. The age of patients with AP associated with AIS was significantly higher compared to those without AP. This correlation may be related to risk factors such as pharyngeal sensory nerve damage and weakened cough reflex which are major causes of aspiration in elderly patients [30]. (2) **NIHSS score**: It had a good predictive effect on the degree of neurological damage, and dysphagia after AIS was closely related to neurological function. Furthermore, stroke patients with a high initial NIHSS score are at an increased risk for poor functional prognosis [31]. (3) **Dysphagia**: Dysphagia could contribute to malnutrition as it impairs the ability to eat and drink, resulting in a decrease in prealbumin levels. Furthermore, if water or food was inadvertently aspirated into the airways and lungs, it could cause obstruction and lead to AP [1]. A meta-analysis revealed a substantial positive correlation between dysphagia and the incidence of AP, with an astounding OR of 9.84 [32]. (4) **Atrial fibrillation**: Studies have indicated that the detachment of mural thrombus resulting from atrial fibrillation was also a significant cause of AIS. Furthermore, a separate study involving 130 hospitalized patients aged over 75 with AP demonstrated that atrial fibrillation significantly reduced the 90-day survival rate of patients (P = 0.039) [33]. (5) There were also studies reporting that patients with poor organ function (such as **Cardiac, Renal,** and **Hepatic insufficiency**) and overall nutritional status (**Prealbumin**) caused by long-term disease status might also have an increased susceptibility to AP [34]. (6) **FBG**: During a serious infection, the body undergoes a heightened state of stress leading to the secretion of various stress hormones that cause elevated blood sugar levels. This, in turn, further exacerbated metabolism and worsened the condition, forming a positive feedback loop for high blood sugar. Additionally, increased blood sugar levels could weaken the body's resistance to infection [35]. Moreover, individuals with diabetes, who already experienced metabolic disorders and compromised immune function, were more susceptible to developing difficulties in swallowing and delayed esophageal emptying, which subsequently increased the risk of AP. In a cohort study involving 89 patients, a higher prevalence of esophageal motility disorders was observed in diabetic patients compared to non-diabetic patients (60 % versus 29.6 %) [36]. (7) **CRP and NEUT%**: As a mixed infection, there was a large number of pathogen proliferation in the body. Even when the symptoms were not obvious at early stages, there could be an increase in infection-related indicators such as CRP and NEUT% [37].

We created a model using selected risk factors to predict AP after AIS. This model is presented in the form of a nomogram. The nomogram model had a C-index of 0.872 (95 % CI: 0.845–0.899) in the training group, 0.847 (95 % CI: 0.800–0.894) in the validation group, and 0.865 (95 % CI: 0.841–0.889) overall, which indicated that the nomogram had a good predictive value. Good predictive efficiency could assist in finding the high-risk patients, so as to give the corresponding support and anti-infection treatment immediately, which could minimize the frequency of adverse prognosis. Nomograms could visually reflect the quantified score for each risk factor and allow to calculate of the predictive values for individual outcome events and the corresponding degree of risk by summing the total points up [38].

Our study had several notable advantages. First, as we know, this was the first retrospective cohort study to utilize a validated nomogram tool for predicting AP in patients with AIS. This innovative approach strengthens the reliability of our findings Second, our study encompassed a large clinical cohort, providing robust evidence and addressing the research gap regarding factors influencing AP in AIS patients. However, despite these advantages, we acknowledge several limitations. Firstly, our research is limited by insufficient authorization to access and combine data from multiple centers for analysis. The retrospective design and reliance on data from a single center introduce inherent biases that may impact the validity of our results. To overcome these limitations, we plan to use data from multiple centers in China for future research to improve the generalizability of our findings. Secondly, our analysis of risk factors was not comprehensive as smoking, a well-established risk factor for stroke that triples mortality, couldn't be included due to challenges in the standardized collection of admission medical histories [39]. We will focus on scientific collection and management of medical history in future studies.

Regarding the mechanism by which smoking promotes the occurrence of AP in AIS patients, several hypotheses have been proposed. Smoking is known to impair lung function and weaken the immune system, making individuals more susceptible to respiratory infections such as aspiration pneumonia (AP) [40]. A cohort study of 2295 adolescents aged 16 showed that exposure to a smoking environment was associated with a 1.1 % decrease (95 % CI -2.0 % to −0.2 %) in FEV1/FVC [41]. Another recent review pointed out that smoking exerts multifaceted effects on alveolar macrophages, including changes in phenotype, phagocytic and bactericidal activities, ROS production, protease/antiprotease release, alterations in iron and lipid homeostasis, as well as changes in exosome secretion [42]. Moreover, smoking damages the cilia and mucociliary clearance in the respiratory tract, leading to the accumulation of mucus and pathogens in the lungs. Based on animal experiments using bullfrogs, it was found that cigarette smoke significantly reduced the mucociliary transport velocity (from 0.14 ± 0.03 mm/s to 0.00 mm/s) [43]. This creates an environment favorable for bacterial growth and increases the risk of AP. In addition, smoking-induced inflammation and oxidative stress can impair the integrity of the airway epithelium and weaken the defense mechanisms against infection [44]. This further enhances the likelihood of developing AP in AIS patients who smoke. Encouragingly, research has found that LL-37 can counteract the effects of cigarette smoke extract on the epithelial tight junction proteins in the airway [45]. It is important for future studies to include smoking as a risk factor and further elucidate the underlying mechanisms by which smoking contributes to the development of AP in AIS patients. By understanding these mechanisms, we can implement targeted interventions and preventive strategies to reduce the incidence of AP in this specific patient population.

What's more, our future research should explore the potential role of genetic factors in predicting the risk of AP after AIS. Investigating genetic variations associated with inflammation (like NOD1, APC, etc.) and immune response could uncover novel biomarkers for risk prediction and personalized interventions [46,47]. Specific genes, such as Toll-like receptors (TLRs) and cytokines, have been implicated in modulating inflammatory processes [48]. It was reported that a panel consisting of IL-12, IP-10, and IL-6 could predict the occurrence of AP after AIS with relatively high accuracy (AUC: 0.9) [49]. Integrating genetic markers into nomogram models may improve risk prediction accuracy and allow for personalized preventive measures, which need further validation through large-scale studies to substantiate. Understanding the genetic basis of AP after AIS could enhance our understanding of disease mechanisms and guide targeted interventions for high-risk individuals.

Therefore, while our study provides valuable insights, it is important to interpret the results cautiously due to the aforementioned limitations. Future research should aim to address these limitations by including comprehensive risk factors and external validation of the predictive model, while also investigating the specific mechanisms through which smoking promotes the occurrence of AP in AIS patients.

## 5 Conclusions

In summary, age, NIHSS score, dysphagia, atrial fibrillation, cardiac insufficiency, renal insufficiency, hepatic insufficiency, FBG, CRP, NEUT% and prealbumin were independent factors associated with the development of AP in AIS patients. The nomogram model's predicted results, which were based on the independent risk factors mentioned above, aligned well with the actual outcomes. So it could provide reference value for the identification of AIS-associated AP in clinical practice and is of great significance for the early adoption of effective prevention and treatment measures, which could improve patient prognosis.

## Funding statement

This work was supported by grants from the 10.13039/100017950Shanghai Municipal Health Commission (Grant No. 202040174) and the 10.13039/501100003399Science and Technology Commission of Shanghai Municipality (Grant No. 22Y11900800).

## Data availability statement

The data used in our study has not been deposited into a publicly available repository due to the authors not having permission to share the data. Additional data and materials may be requested from the corresponding author upon reasonable request.

## Additional information

No additional information is available for this paper.

## CRediT authorship contribution statement

**Junming Wang:** Conceptualization, Formal analysis, Methodology, Visualization, Writing – original draft, Writing – review & editing. **Yuntao Wang:** Conceptualization, Data curation, Investigation, Validation, Writing – review & editing. **Pengfei Wang:** Data curation, Investigation, Validation, Writing – review & editing. **Xueting Shen:** Data curation, Investigation, Resources, Writing – review & editing. **Lina Wang:** Data curation, Investigation, Resources, Writing – review & editing. **Daikun He:** Funding acquisition, Investigation, Project administration, Resources, Supervision, Validation, Writing – original draft, Writing – review & editing.

## Declaration of competing interest

The authors declare that they have no known competing financial interests or personal relationships that could have appeared to influence the work reported in this paper.

## Abbreviations

AP: Aspiration Pneumonia
AIS: Acute Ischemic Stroke
NIHSS: National Institute of Health Stroke Scale
SAP: Stroke Associated Pneumonia
COPD: Chronic Obstructive Pulmonary Disease
CHD: Coronary Heart Disease
FBG: Fasting Blood-Glucose
CRP: C-Reactive Protein
WBC: White Blood Cell count
NEUT%: Neutrophilic Granulocyte Percentage
HB: Hemoglobin
PLT: Platelet Count
TC: Total Cholesterol
LDL: Low-Density Lipoprotein
K^+^: Potassium
Na^+^: Sodium
ROC: Receiver Operating Characteristic
AIS-APS: Acute Ischemic Stroke Associated Pneumonia Score
