# Supplementary material for: Construction and evaluation of a nomogram prediction model for aspiration pneumonia in patients with acute ischemic stroke
Source: Heliyon. 2023 Nov 8;9(11):e22048. doi: 10.1016/j.heliyon.2023.e22048 (PMC10682132; doi:10.1016/j.heliyon.2023.e22048)
Supplement: Supplementary file 1 [file mmc1.docx]

Suppl. Table 1. Description of Variable Assignment.

| **Variables** | **Factors** | **Assignment Description** |
| --- | --- | --- |
| ***X1*** | Age (y) | ≤44=0; 45-59=1; 60-74=2; 75-89=3; ≥90=4 |
| ***X2*** | Gender | Female=0; Male=1 |
| ***X3*** | NIHSS score | ≤5=0, 6-20=1, ≥21=2 |
| ***X4*** | Dysphagia | No=0; Yes=1 |
| ***X5*** | Hyperlipidemia | No=0; Yes=1 |
| ***X6*** | COPD | No=0; Yes=1 |
| ***X7*** | CHD | No=0; Yes=1 |
| ***X8*** | Atrial fibrillation | No=0; Yes=1 |
| ***X9*** | Cardiac insufficiency | No=0; Yes=1 |
| ***X10*** | Renal insufficiency | No=0; Yes=1 |
| ***X11*** | Hepatic insufficiency | No=0; Yes=1 |
| ***X12*** | FBG (mmol/L) | 4-7=0; <4 or >7=1 |
| ***X13*** | CRP(mg/L) | <10=0; 10-19=1; 20-29=2; 30-39=3; 40-49=4; ≥50=5 |
| ***X14*** | WBC (×109/L ) | 4-10=0; <4 or >10=1 |
| ***X15*** | NEUT%(%) | <80=0; ≥80=1 |
| ***X16*** | HB(g/L) | ≥120=0; 119-90=1; 89-60=2; <60=3 |
| ***X17*** | Albumin (g/L) | ≥40=0; 39-30=1; <30=2 |
| ***X18*** | Prealbumin (mg/L) | ≥180=0; 179-150=1; 149-100=2; <100=3 |
| ***X19*** | Triglyceride (mmol/L) | 0.56-1.7=0; <0.56 or >1.7=1 |
| ***X20*** | K^+^(mmol/L) | 3.5-5.0=0; <3.5 or >5.0=1 |
| ***Y*** | AP | NO=0; Yes=1 |

Note. COPD=Chronic Obstructive Pulmonary Disease; CHD=Coronary Heart Disease; FBG=Fasting Blood-Glucose; CRP=C-reactive Protein; NEUT%=Neutrophilic Granulocyte Percentage; WBC=White Blood cell Count; HB=Hemoglobin; PLT=Platelet Count; K^+^=Potassium.
